# Meta-Analysis of the Clinical Effectiveness and Safety of Ligustrazine in Cerebral Infarction

**DOI:** 10.1155/2016/3595946

**Published:** 2016-09-21

**Authors:** Tian Yu, Xiaoheng Guo, Zhen Zhang, Rong Liu, Liang Zou, Jia Fu, Zheng Shi

**Affiliations:** Chengdu University College of Medicine, Chengdu, China

## Abstract

*Objectives*. To evaluate the efficacy and safety of ligustrazine in the treatment of cerebral infarction.* Methods*. A systematic literature search was conducted in 6 databases until 30 June 2016 to identify randomized controlled trials (RCTs) of ligustrazine in the treatment of cerebral infarction. The quality of all the included studies was evaluated. All data were analyzed by Review Manager 5.1 Software.* Results*. 19 RCTs totally involving 1969 patients were included. The primary outcome measures were Neurological Deficit Score (NDS) and clinical effective rate. The secondary outcome measure was adverse events. Meta-analysis showed that ligustrazine could improve clinical efficacy and NDS of cerebral infarction with [OR = 3.60, 95% CI (2.72, 4.78), *P* < 0.00001] and [WMD = −3.87, 95% CI (−4.78, −2.95), *P* < 0.00001]. Moreover, ligustrazine in treatment group exerted better clinical effects in improving the Blood Rheology Index (BRI) in patients compared with control group. Ten trials contained safety assessments and stated that no obvious side effects were found.* Conclusions*. Ligustrazine demonstrated definite clinical efficacy for cerebral infarction, and it can also improve NDS in patients without obvious adverse events. However, due to the existing low-quality research, more large-scale and multicentric RCTs are required to provide clear evidence for its clinical efficacy in the near future.

## 1. Introduction

Cerebral infarction is a disease of ischemia which commonly causes the brain tissue necrosis softening, and the patients often exhibit corresponding nervous system symptoms, such as hemiplegia and aphasia. It is reported that China has the highest death rate of cerebral vascular disease around the world. The incidence rate of cerebral infarction in stroke is around 50%~60%, ranked first in the world [1]. Hitherto, a number of clinical drugs were used for treatment of cerebral infarction. And, it is known to all that Chinese Patent Medicine (CPM) has been used in clinical practice for 30 years, and it has been approved by the Chinese State Food and Drug Administration for clinical trials [2]. Therefore, evaluation of the effectiveness and safety of Chinese Patent Medicine (CPM) could have a great impact on cerebral infarction management in the world.

**Figure 1:**
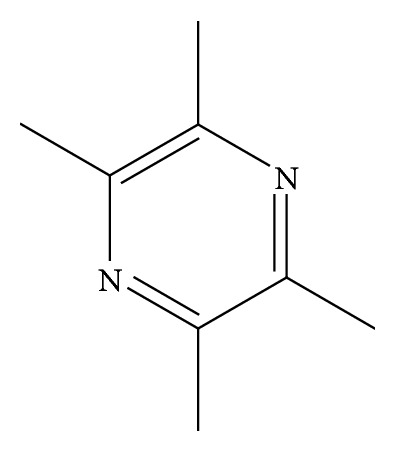
Structure of ligustrazine.

Accumulating studies have been demonstrated on the prevention and treatment of cerebral infarction by CPM in China, and some progress has been made, most notably by using ligustrazine. Ligustrazine (tetramethylpyrazine, short form TMP) is a bioactive ingredient extracted from a widely used Chinese herb, Chuanxiong (*Rhizoma Chuanxiong*) [3]. The content of ligustrazine in Chuanxiong is only 0.01%~0.02%, and its chemical structure is tetramethylpyrazine (Figure 1) [4]. According to the compendium of Materia Medica records, Chuanxiong is spicy in taste and warm in nature and belongs to the hepatobiliary pericardium, which has the effect of promoting blood circulation of Qi, dispelling wind, and relieving pain [5].

As one of the most attention-getting CPMs, ligustrazine is well studied for its multiple significant biological functions, such as inhibiting apoptosis, dilating blood vessels, protecting vascular endothelial cells and immune regulation, eliminating oxygen free radical, improving cerebral ischemia, inhibiting platelet aggregation, and promoting angiogenesis [6–8]. Ligustrazine is an inhibitor of phosphodiesterase, which has been widely used for treatment of cardiovascular diseases in China [9]. Modern pharmacological experimental studies showed that ligustrazine plays a vital role in antithrombosis* in vivo*, the mechanism seems to be related to the inhibition of platelet aggregation and protection of endothelium [10]. Ligustrazine injection combined with Buyanghuanwu decoction can improve blood flow, promote the neurological function in patients, and improve the clinical efficacy in the treatment of cerebral infarction which is caused by blood stasis due to Qi deficiency [11]. Ligustrazine is a new type of calcium ion antagonist and free radical scavenger, which can pass through the blood brain barrier. High dose of ligustrazine can significantly enhance the expression of Bcl-2 and reduce the expression of p53 [12]. Moreover, ligustrazine can attenuate cerebral ischemia reperfusion injury and the protective mechanism may be partly through regulating caspase-12 gene expression and reducing neuronal apoptosis [13].

Currently, the mechanisms of ligustrazine in the treatment of cerebral infarction were revealed by a large number of animal pharmacological experiments. However, the statistical analysis of the clinical efficacy and safety evaluation is still lacking. Herein, the aim of this study is to evaluate the effectiveness and safety of ligustrazine in the prevention and treatment of cerebral infarction. As a result, ligustrazine demonstrated definite clinical efficacy for cerebral infarction, and it also improved NDS in patients without obvious adverse events.

## 2. Materials and Methods

### 2.1. Search Strategy

First, we searched CNKI (1979 to 30 June 2016), VIP (1989 to 30 June 2016), Cochrane Library (1947 to 30 June 2016), Medline (1905 to 30 June 2016), PubMed (1947 to 30 June 2016), and Academic Search Premier (1950 to 30 June 2016), using the search terms “cerebral infarction” AND “ligustrazine” or “tetramethylpyrazine” or “TMP” or “chuanxiongqin” AND “randomized controlled trial” or “RCT”. Subsequently, we retrieved further information by using manual retrieval of references from recent reviews and relevant published original studies.

### 2.2. Inclusion Criteria

All the experiments are designed to follow the RCT plan, whether or not the blind method or the allocation concealment is mentioned. Participants diagnosed with cerebral infarction according to Western medicine diagnostic standard to the Diagnosis of Cerebral Vascular Diseases (DCVD) were included, regardless of gender, age, or ethnicity. In addition, CT scan or MRI was applied to confirm diagnosis of cerebral infarction. On the basis of conventional treatment, the treatment group was treated with ligustrazine alone or in combination with other routine treatments, while the control group was treated with conventional therapy, and the two groups except for the treatment of ligustrazine were relatively consistent with other treatments. The balance among the groups was better and the basic information of the patients was not statistically significant.

### 2.3. Exclusion Criteria

Exclusion criteria are as follows: combined cerebral hemorrhage and other serious organic diseases or complications; the research object being the animal or tissue cell; the same clinical research literature being published in different journals; the results of the variables used in the original literature being inconsistent with the other documents; nonrandomized controlled trial; review and conference summary.

### 2.4. Data Extraction and Quality Assessment

Two investigators (Tian Yu and Xiaoheng Guo) independently abstracted data from eligible studies. Discrepancies were resolved by discussion with a third investigator (Zhen Zhang) and by referencing the original report. Literature quality evaluation is utilized by Cochrane system manual. At the same time, researchers took advantage of Jadad quality score method to evaluate the quality of the included studies, including whether to describe the random method, whether to use blind method, and whether to report allocation concealment. Using 5-point scoring method, 1 point for each aspect, 1~3 represent low-quality literature and 4~5 high quality literature. Relevant data included the first author's name, study name, year of publication, number of participants, age of participants, course of treatment, course of disease, interventions, outcome assessment, and corresponding 95% confidence interval.

### 2.5. Statistical Analysis

We utilized Cochrane Collaboration's Review Manager Software Package (RevMan 5) for the meta-analysis of observational studies. Clinical heterogeneity analysis of the included studies was conducted by *χ* ^2^ test. If *P* > 0.05, *I* ^2^ < 50%, there was no statistical heterogeneity between the results of the study and meta-analysis was used by fixed effect model; otherwise random effects model was selected. If heterogeneity is too large to be analyzed by Meta, then descriptive analysis was further applied. Moreover, researchers took advantage of a funnel plot analysis to analyze the possible publication bias. The categorical data were analyzed by using odds ratio (OR) and the measurement data were analyzed through weighted mean difference (WMD) approach.

## 3. Results

### 3.1. Study Screening Process

**Figure 2:**
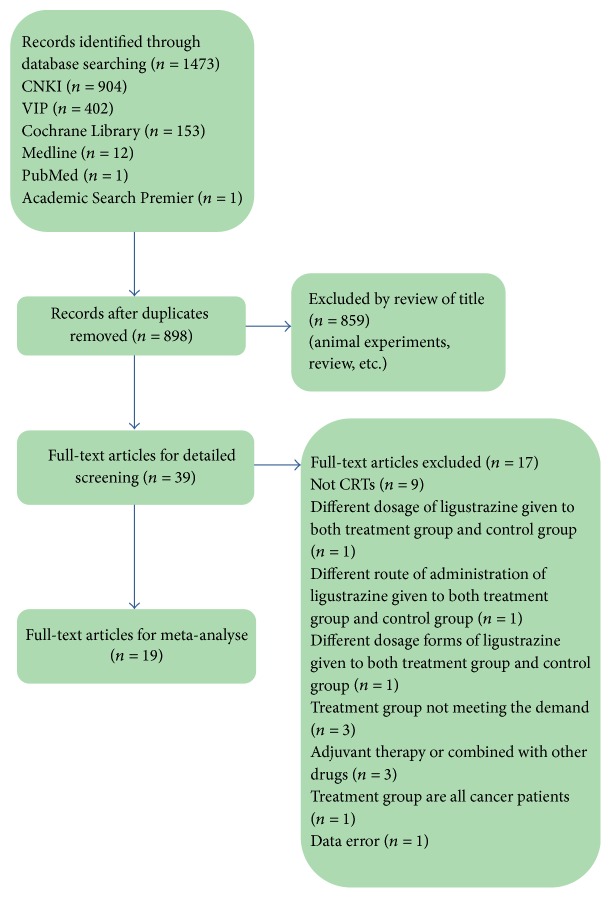
Screen process.

1473 articles were retrieved according to the search strategy and data collection method. Complying with inclusion and exclusion criteria, 19 RCT studies were eventually included after reading the title, abstract, and full text [14–32]. Study screening process and results are shown in Figure 2.

### 3.2. Study Identification and Characteristics

1969 patients (treatment group 999 and control group 970) on 19 RCTs were included; all trials were conducted in China. The baseline conditions including age, gender, and severity of illness were compared between the treatment group and the control group. All included trials applied standard western medicine diagnostic criteria for cerebral infarction. All the studies have reported that the CT/MRI scanning was used for the patients in order to confirm the diagnosis.

In the intervention program, the treatment group consisted of 2 RCTs single with ligustrazine and 17 RCTs in combination with other routine drugs. The control group consisted of 5 RCTs with Danshen injection or compound Danshen injection, 3 RCTs of routine treatment of Western medicine, 2 RCTs with Weinaolutong injection, 2 RCTs with D-40 injection, 1 RCT with Xuesaitong injection, 1 RCT with Weinaolutong injection and citicoline injection, 1 RCT with compound Danshen injection and Troxerutin injection and sodium ozagrel, 1 RCT with Danshen injection and citicoline injection, 1 RCT with Runtan injection, 1 RCT with nimodipine and cerebrolysin, and 1 RCT with ozagrel sodium chloride injection and citicoline and aspirin (shown in Table 1).

*Quality Assessment of Methodology*. The baseline situation of the patients after grouping was reported in 16 studies. The basic information including age, gender, neurological function deficit scale, ischemic area, and course of disease before treatment between different groups has no statistical difference and comparability. Additionally, the methodological quality of most included trials was generally “poor”; only three RCTs described the methods of randomization [22, 26, 27]. The Jadad score of all the studies was 1 point. Moreover, all the studies did not report the number of patients lost to follow-up, and all trials did not mention blinding or intention to treat analysis.

### 3.3. Risk of Bias within Studies

All of the included studies claimed randomization, but only 1 study reported the method of random sequences generation [27]. No study mentioned allocation concealment and blinding procedures. The dropout data were not reported in all of the included studies and selective reporting was found in the majority of the trials.

**Figure 3:**
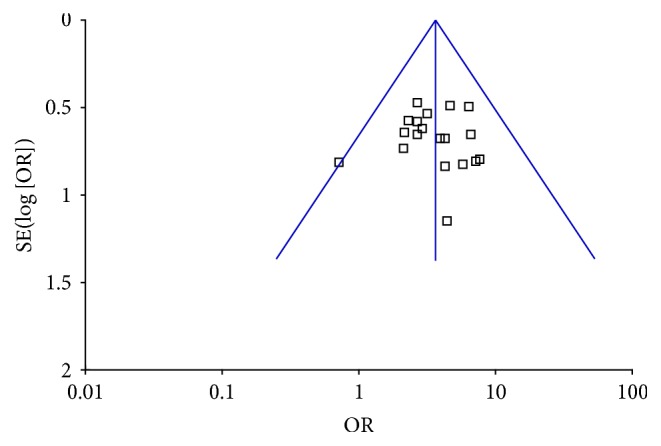
Funnel plot of comparison: meta-analysis of the clinical effectiveness of ligustrazine in Cerebral infarction.

Furthermore, a funnel plot was utilized to analyze the publication bias. The figure shows a general symmetrical funnel shape. There is no obvious publication bias among the studies (Figure 3).

### 3.4. Results of Meta-Analysis

#### 3.4.1. NDS

**Figure 4:**
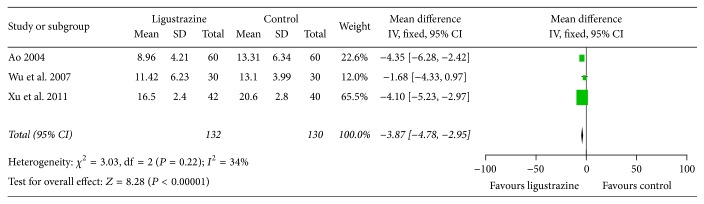
Forest plot of comparison: meta-analysis of the effect of ligustrazine on NDS of cerebral infarction.

5 of the 19 studies reported the comparison of NDS; 4 provided detailed data. Data extracted from these 4 studies [14, 24, 27, 31] showed heterogeneity in the consistency of trial results (heterogeneity: CHI^2^ = 21.45, *P* < 0.05, *I* ^2^ > 86%). The results suggested that the research is highly heterogeneous, and then publication bias analysis was also applied. Moreover, we analyzed the factors which may have a significant impact on the results, such as method of sample selection and determination index. As a result, we found that only one study [24] did not provide the comparison of NDS between two groups before treatment. In order to improve the accuracy of analysis, this reference was excluded. Subsequently, a further meta-analysis of NDS was conducted by using the rest of the three studies [14, 27, 31]. The result showed a low heterogeneity (CHI^2^ = 21.45, *I* ^2^ > 86%), and fixed effects model should be applied. The combined value of WMD was −3.87 (95% CI = −4.78~−2.95). Significance test *Z* = 8.28, *P* < 0.00001. The results showed that ligustrazine had improved the NDS compared with control group (Figure 4).

#### 3.4.2. The Clinical Effective Rate

**Figure 5:**
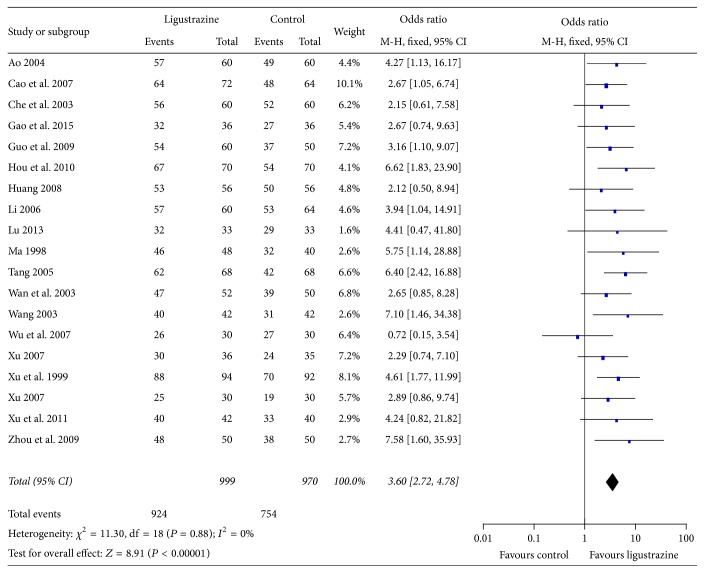
Forest plot of comparison: meta-analysis of the effect of ligustrazine on clinical effects of cerebral infarction.

All 19 included studies adopted the clinical effective rate to assess the clinical improvement. The fixed effect model was used for statistical analysis because of the heterogeneity (heterogeneity CHI^2^ = 11.30, *P* = 0.8, *I* ^2^ = 0% < 50%). The combined effects of 19 independent trial *R* = 3.60 (95% CI = 2.72~4.78). Significance test *Z* = 8.91, *P* < 0.00001. The results showed that ligustrazine had improved the clinical effective rate in patients with cerebral infarction when compared with control group (Figure 5).

#### 3.4.3. BRI

8 of the 19 studies [14, 15, 18, 21, 22, 27, 28, 31] reported the effect of ligustrazine on BRI. The results showed that ligustrazine had improved BRI in patients with cerebral infarction when compared with control group.

### 3.5. Adverse Events

Ten studies [14, 21, 23–26, 28, 30–32] reported the adverse events, while five of them reported minor adverse events. One patient had mild headache and disappeared by slowing drop [14], one patient showed cutaneous eruption and disappeared after withdrawal [23], two patients had mild abdominal discomfort, dizziness, and dry mouth [24], one patient had drowsiness at the beginning of treatment and it released by itself [28], and four patients had mild headache which disappeared after withdrawal [32]. There were no significant differences in the results of blood routine, urine routine, liver functions, renal function, or blood glucose in both groups of patients before and after treatment.

## 4. Discussion

### 4.1. Primary Outcome

Cerebral infarction is a common nervous system disease. Such disease not only causes the decline of life quality in patients but also causes great pressure in medicinal and health service. Therefore, the research and prevention of cerebral vascular disease has become an urgent public health problem in our country, and it is of great importance to use proper treatment for prevention and effective treatment of cerebral infarction.

Western medicine, commonly by using thrombolysis, anticoagulation, fibrinolysis, and cerebral protection, has been used as the main treatment of cerebral infarction in recent years. And it has several advantages such as quick effect and better short-term therapeutic effects. Although tremendous progress has been made by using Western medicine, it has been increasingly recognized that Western medicine is not the best solution in some cases; the main disadvantage is serious side effects [33]. Ligustrazine is commonly used in Traditional Chinese Medicine for activating blood and resolving stasis and the prevention and cure effect of ligustrazine on cerebral infarction have been the concern of clinical workers for a long time.

To the best of our knowledge, it is the first time to report the efficacy and safety by using meta-analysis of ligustrazine for cerebral infarction. Firstly, we took the improvement effect of ligustrazine on NDS, clinical efficacy, and BRI as the main outcome measures; meta-analysis was performed with control group without ligustrazine. Subsequently, nineteen studies with 1969 individuals suffering from cerebral infarction were selected. The main finding of present study was that ligustrazine could improve NDS of cerebral infarction (Figure 4). It also can be seen from Figure 5 that ligustrazine had improved the clinical effective rate in patients with cerebral infarction compared with control group. Moreover, ligustrazine seems generally safe, but it is hard to make a conclusion on the issue of safety because there are only 52.6% studies that mentioned the adverse events.

Notably, the main side effects by using ligustrazine are mild headache, abdominal distention, nausea, and so forth. There are ten studies that reported the adverse events, and five of them clearly reported minor adverse events. However, some RCTs did not report adverse events, and all RCTs did not conduct statistical analysis of adverse events between the two groups. No specific data can support the results of adverse events. After a comprehensive analysis, we can only conclude through the present evidence that the adverse events rate in the process of the use of ligustrazine is relatively low. And there is not enough compelling quantitative statistical data supporting its safety.

In this article, the concentrations of ligustrazine used in each trial are different; there are two main reasons. First, the patients' condition in each clinical trial is different, so different doses of ligustrazine were taken according to the severity of cerebral infarction. Second, ligustrazine used in each clinical trial was from different manufacturers. Therefore, the agents with different specifications and dosage guidance were applied to the patients. So far, there is no uniform clinical medication dose of ligustrazine in the treatment of cerebral infarction.

### 4.2. Limitations

Firstly, it is necessary to use the randomization to avoid selection bias. In this work, there was only 1 study that provided specific information on how the random allocation was generated. The allocation concealment was not reported in all included trails. Excessively higher estimate of treatment effect could be caused by inadequate allocation concealment. Secondly, blinding and placebo control were not mentioned in all studies. After completing the treatment, the efficacy was evaluated immediately. The long-term effect of ligustrazine treatment cannot be evaluated due to the lack of follow-up. Finally, sample sizes in individual trial are relatively small and this may cause a reduction of reliability in statistical analysis.

## 5. Conclusions

Totally 1969 articles were retrieved according to the search strategy and data collection methods, and finally there were 19 studies included that comply with inclusion and exclusion criteria, for the meta-analysis of ligustrazine in cerebral infarction. The results indicated that ligustrazine had improved the clinical effective rate and NDS in patients with cerebral infarction when compared with control group. Ten studies reported the adverse events; the results showed that the incidence of adverse events was low and slight; ligustrazine in the treatment of cerebral infarction is safe. However, current evidence is insufficient to support the efficacy of ligustrazine for cerebral infarction because the included studies were of generally poor quality and small sample sizes. Therefore, it is necessary to carry out multicenter, large sample, high quality double-blinded randomized controlled trials in future research.

## Figures and Tables

**Table 1:** Characteristics of the included studies.

Included studies	PY	EC	CD (h)		Study design	Sample size (M/F)				Ave. age (range or SD)		CM (d)	Interventions		Outcomes	IF
			TRE	CON		TRE (M/F)		CON (M/F)		TRE	CON		TRE	CON		
Ao [14]	2004	DCVD1995	NA	NA	RCT	60	(46/14)	60	(47/13)	36–78 (63.11 ± 8.22)	NA	14 × 2	LI 160–200 mg qd	Xuesaitong 400 mg qd	NDS	*P* < 0.01
															BRI	*P* < 0.05
															Clinical efficacy	*P* < 0.05

Cao et al. [15]	2007	DCVD1995	<72	<72	RCT	72	(48/24)	64	(42/22)	49–75 (62.2 ± 12.7)	48–75 (61.6 ± 13.4)	14	LHI 300 mg qd	RTWM	BRI	*P* < 0.05
															TNF-*α* level	*P* < 0.05
															Clinical efficacy	*P* < 0.05

Che and Yang [16]	2003	DCVD1995	57.6 ± 12	60 ± 9.6	RCT	60	(40/20)	60	(36/24)	54 ± 5	55 ± 4	15	LI 200 ml qd	WeinaoLutong injection 0.4 g + citicoline injection 0.5 g qd	Clinical efficacy	*P* < 0.05

Gao [17]	2015	DCVD1995	NA	NA	RCT	36	(17/19)	36	(21/15)	47–76 (60.4 ± 8.6)	48–78 (59.5 ± 7.5)	14	LI 10 ml qd	Compound Danshen injection 20 ml qd	Clinical efficacy	*P* < 0.05

Guo and Yang [18]	2009	DCVD1995	<72	<72	RCT	60	(38/22)	50	(34/16)	48–78 (59.3 ± 10.4)	49–76 (57.9 ± 13.1)	14	LHI 80 mg qd	Compound Danshen injection 20 ml qd	BRI	*P* < 0.05
															NDS	*P* < 0.05
															Clinical efficacy	*P* < 0.05

Hou [19]	2010	DCVD1995	NA	NA	RCT	70	(40/30)	70	(38/32)			20	LI 160 mg bid	Compound Danshen injection 20 ml + Troxerutin injection 0.6 g + sodium ozagrel 250 ml qd	Clinical efficacy	*P* < 0.05

Huang [20]	2008	DCVD1996	NA	NA	RCT	56	(31/25)	56	(33/23)	63.7	63.9	14	LI 160 mg qd	WeinaoLUtong injection 600 mg qd	Clinical efficacy	*P* < 0.05

Li [21]	2006	DCVD1996	<48	<48	RCT	60	NA	64	NA	62.8 ± 2.4	NA	14	LI 80 mg qd	Danshen injection 250 ml qd. + citicoline injection 1.0 g qd	NIHSS	*P* < 0.01
															BRI	*P* < 0.05
															Clinical efficacy	*P* < 0.05

Lu [22]	2013	DCVD1996	<24	<24	RCT	33	NA	33	NA	55–76 (65.2 ± 12.3)	NA	14	LI 80 mg qd	RTWM	BRI	*P* < 0.05
															Clinical efficacy	*P* < 0.05

Ma [23]	1998	DCVD1996	<25	<20	RCT	48	(33/15)	40	(27/13)	49–80 (62)	47–85 (64)	14	LI 200 mg qd	D-40 500 ml qd	Muscle strength recovery time	*P* < 0.05
															Clinical efficacy	*P* < 0.05

Wan et al. [24]	2003	DCVD1996	<120	<168	RCT	52	(29/23)	50	(30/20)	52–83 (62.7)	48–78 (63.2)	14	LHI 80 mg qd	Danshen injection 16 ml qd.	NDS	*P* < 0.05
															Clinical efficacy	*P* < 0.05

Tang [25]	2005	DCVD1995	2–46	2–42	RCT	68	(46/22)	68	(44/24)	48–78 (68)	53–77 (63)	14	LI 160 mg qd	Routine treatment of Western medicine	Clinical efficacy	*P* < 0.01

Wang [26]	2003	DCVD1999	NA	NA	RCT	42	NA	42	NA	39–83 (62)	NA	10 × 2	LI 200 mg qd	WeinaoLutong injection 0.5 g qd	Clinical efficacy	*P* < 0.05

Wu and Ding [27]	2007	DCVD1995	<24	<24	RCT	30	(18/12)	30	(16/14)	63.32	65.23	21	LI 160 mg qd	Runtan injection 20 mg qd	BRI	*P* < 0.05
															NDS	*P* < 0.01
															GCS	*P* < 0.01
															Clinical efficacy	*P > *0.05

Xu [28]	2007	DCVD1996	<72	NA	RCT	36	(24/12)	35	(24/11)	56–78 (62.7)	53–81 (63.8)	14	LHI 80 mg bid	Compound Danshen injection 20 ml qd	BRI	*P* < 0.05
															Clinical efficacy	*P* < 0.05

Xu [29]	2007	DCVD1995	NA	NA	RCT	30	(19/11)	30	(17/13)	56–82 (69.2)	55–83 (68.7)	14	LI 100 mg qd	Danshen injection 16 ml qd	BI	*P* < 0.05
															Clinical efficacy	*P* < 0.05

Xu et al. [30]	1999	DCVD1986	72–168	NA	RCT	94	(58/36)	92	(56/36)	41–76 (59.6)	43–75 (58.8)	12	LI 600–800 mg qd	Nimodipine 4 mg i.v.qd + cerebrolysin 10 ml	Clinical efficacy	*P* < 0.01

Xu et al. [31]	2011	DCVD1995	6–72	NA	RCT	42	(24/18)	40	(24/16)	56–78 (62.8 ± 2.5)	55–81 (63.2 ± 2.7)	14	LHI 120 mg qd + ozagrel sodium chloride injection 100 ml qd + Citicoline 1.0 g qd + Asiprin 100 mg qd	Ozagrel sodium chloride injection 100 ml qd + citicoline 1.0 g qd + Aspirin 100 mg qd	BRI	*P* < 0.05
															NDS	*P* < 0.05
															Clinical efficacy	*P* < 0.05

Zhou and Zhao [32]	2009	DCVD1995	NA	NA	RCT	50	NA	50	NA	NA	NA	14	LHI 100 mg qd + CT	D-40 500 ml qd + CT	Clinical efficacy	*P* < 0.01

PY: publication year; TRE: treatment; CON: control; M: male; F: female; Ave.: average; EC: eligibility criteria; CD: course of disease; CM: course of treatment; DCVD: diagnosis of cerebral vascular diseases; RCT: randomized controlled trial; d: day (s); h: hour (s); NDS: neurological deficit score; CT: conventional therapy (nimodipine + xuesaitong + cerebrolysin); LI = ligustrazine injection; LHI = ligustrazine hydrochloride injection; BRI: blood rheology index; BI: Barthel Index; GCS: Glasgow coma scale; RTWM: routine treatment of Western medicine (dilate blood vessel; reduce intracranial pressure; control blood pressure, blood glucose, and blood lipid; prevent infection; correct the disorder of water and electrolyte; maintain acid-base balance, etc.); NIHSS: national institutes of health stroke scale; TL: TNF-*α* level; CE: clinical efficacy; MSRT: muscle strength recovery time; NA: not available.
